# Ten-year outcomes of pulmonary endarterectomy in Switzerland: The Zurich experience

**DOI:** 10.1016/j.xjon.2025.11.025

**Published:** 2025-12-01

**Authors:** Bianca Battilana, Kathrin Chiffi, Tobias Renner, Rea Andermatt, Thomas Frauenfelder, Milan Miladinovic, Monika Hebeisen, Gilbert Puippe, Mona Lichtblau, Reto Schüpbach, Dominique Bettex, Silvia Ulrich, Isabelle Opitz

**Affiliations:** a Department of Thoracic Surgery, University Hospital Zurich, Zurich, Switzerland; b University of Zurich, Zurich, Switzerland; c Institute of Anesthesiology, University Hospital Zurich, Zurich, Switzerland; d Institute of Intensive Care Medicine, University Hospital Zurich, Zurich, Switzerland; e Institute of Diagnostic and Interventional Radiology, University Hospital Zurich, Zurich, Switzerland; f Department of Biostatistics, University Hospital Zurich, Zurich, Switzerland; g Institute of Epidemiology, Biostatistics, and Prevention, University Hospital Zurich, Zurich, Switzerland; h Department of Pulmonology, University Hospital Zurich, Zurich, Switzerland

**Keywords:** chronic thromboembolic pulmonary hypertension, pulmonary endarterectomy, surgical treatment, CTEPH, PEA, benchmark analysis

## Abstract

**Objective:**

Pulmonary endarterectomy (PEA) is the gold standard for operable chronic thromboembolic pulmonary hypertension (CTEPH), an often underdiagnosed and undertreated disease. Before 2015, Swiss patients had limited access to PEA and were operated abroad, highlighting the need for a CTEPH center in Switzerland despite its small population. We herein summarize our 10-year PEA experience, its influence on patient outcomes, and analyze potential prognosticators for complications and long-term outcomes.

**Methods:**

Prospectively collected records of patients with CTEPH undergoing PEA at our institution (January 2015-December 2024) were retrospectively analyzed for perioperative and long-term outcome parameters, prognosticators for complications, and hemodynamic improvement. A benchmark analysis compared our center's results with the International CTEPH Registry.

**Results:**

Our cohort included 141 patients with CTEPH undergoing PEA, with 85 (60.3%) male patients and a median age of 62 years (range, 51-71 years). We observed significant improvements in mean pulmonary arterial pressure (mean difference, 16.6 mm Hg; *P* < .0001), pulmonary vascular resistance (mean difference, 3.7 WU; *P* < .0001), 6-minute walk test (mean difference, 68.8 m; *P* < .0001), oxygen requirement (χ^2^ = 6.3%; *P* = .018), New York Heart Association functional classification (rank difference statistic = −8%; *P* < .0001), and quality of life (Lin coefficient = 13.7 points; *P* = .004) after PEA. In-hospital and 90-day mortality were 2.8% (n = 4). Jamieson IV (odds ratio, 4.22; *P* = .039) and N-terminal pro B-type natriuretic peptide (odds ratio, 1.5; *P* = .039) were associated with postoperative complications. A stronger immediate postoperative decrease in mean pulmonary arterial pressure (mean difference, 0.7 mm Hg; *P* < .0001) and pulmonary vascular resistance (mean difference, 0.4 WU; *P* < .0001) predicted better long-term hemodynamic outcomes. Benchmark analysis showed comparable results with International CTEPH Registry data.

**Conclusions:**

Establishing a PEA program in Switzerland enabled timely, gold standard care for patients with CTEPH. Despite being a small-volume program, outcomes were comparable with high-volume centers. N-terminal pro B-type natriuretic peptide, Jamieson IV, and initial hemodynamic improvements emerged as prognosticators, warranting prospective validation.

## Graphical Abstract

**Figure:**
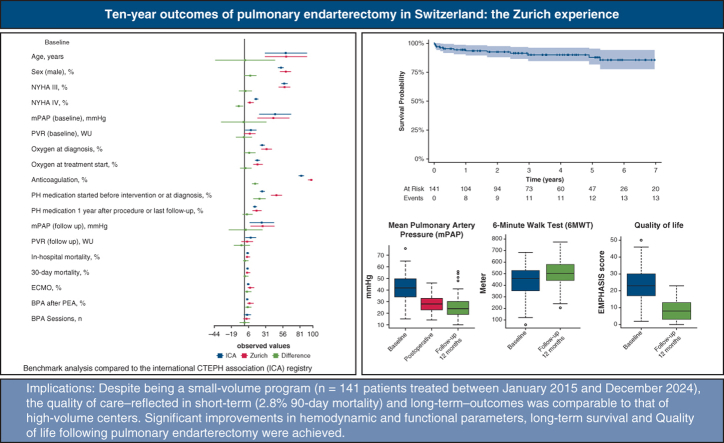

**Figure:**
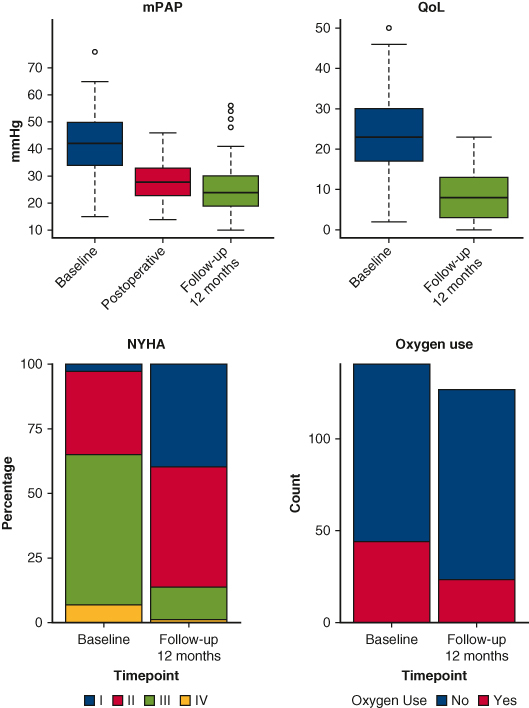
Improvements after PEA in patients with CTEPH.

**Central Message**

This article presents 10-year outcomes from the Zurich PEA program, benchmarks them against the International CTEPH Registry, and analyzes prognostic factors for postoperative outcomes.

**Perspective**

The establishment of a PEA program in a small country like Switzerland offers unique insights into addressing local health care challenges while striving to deliver high-quality care for patients with CTEPH. Highlighting potential new prognosticators for short- and long-term outcomes may support patient stratification, pending confirmation in independent validation studies.

Chronic thromboembolic pulmonary hypertension (CTEPH)—a rare, debilitating precapillary disease, classified as group 4 PH—typically arises as a complication following acute pulmonary embolism and is defined as symptomatic PH with persistent pulmonary perfusion defects despite 3 to 6 months of therapeutic anticoagulation.^1^^,^^2^

Pulmonary endarterectomy (PEA), which involves surgical removal of intraluminal thromboembolic and fibrotic material, remains the only curative treatment for CTEPH.^2^ In experienced centers, operative mortality rates are <5%.^3^ PEA significantly improves medium-to long-term survival and helps maintain good functional status compared with conservative management.^1^^,^^4^

Until 2015, the absence of a PEA program in Switzerland meant patients had to undergo surgery abroad. Consequently, from 2000 to 2012, only 14% of patients with CTEPH received PEA,^5^ compared with a 58% resection rate reported by the International CTEPH Registry.^6^ This highlighted the need for a surgical CTEPH center in Switzerland, despite its small population. Therefore, the first and only PEA program was established in Zurich. We hypothesize that a high-quality PEA program at a low-volume, single national center can achieve outcomes comparable to those reported by larger international registries. We summarize our 10-year experience and its influence on patient outcomes, comparing results to the International CTEPH Registry.^4^^,^^6^ Secondarily, we aim to analyze prognostic factors for hemodynamic improvement, postoperative complications, and need for multimodal treatment following PEA.

## Materials and Methods

### Ethical Statement

All patients were treated at the University Hospital of Zurich and provided informed consent in accordance with ethical approval (BASCE-No. 2020-02566), granted on February 9, 2021.

### Patients and Methods

This retrospective study analyzed all prospectively documented patients with CTEPH undergoing PEA at our institution between January 2015 and December 2024. In 2018, a national multidisciplinary CTEPH Board was established to review cases before surgical referral.^7^ PEAs were performed as previously described.^8^ Patient data were extracted from electronic medical records and managed using REDCap, hosted at the University Hospital of Zurich. Preoperative characteristics, perioperative data, and postoperative follow-up were prospectively recorded. Postoperative complications—such as right ventricular failure, atrial fibrillation, atrioventricular nodal reentry tachycardia, pericardial effusion, cardiac tamponade, lung reperfusion edema, ventilator-associated pneumonia, intrapulmonary bleeding, acute respiratory distress syndrome, respiratory failure, hemothorax, pneumothorax, pulmonary embolism, acute renal failure, ischemic bowel, sternal instability, and wound infection—were included if classified as Clavien-Dindo ≥III. Patients were followed up at 1, 3, 6, and 12 months post-PEA. Follow-up included transthoracic echocardiography (TTE), 6-minute-walk-test (6MWT), and EmPHasis quality of life (QoL) questionnaire at 3, 6, and 12 months; computed tomography angiography at 6 months; and right heart catheterization (RHC) at 12 months. Patients with persistent symptomatic PH and pulmonary vascular resistance (PVR) >8 WU postoperatively received PH-targeted therapy or, in cases of distal vascular obstruction, were considered for balloon pulmonary angioplasty (BPA) as previously reported.^7^

### Statistical Methods

Descriptive statistics were used to summarize continuous variables as mean ± SD or median (interquartile range); categorical variables were presented as count and percentage. Missing values were not imputed; analyses were based on complete cases. Kaplan-Meier analysis was used to assess survival and reverse Kaplan-Meier analysis to assess length of follow-up. A benchmark analysis compared Zurich data with the international CTEPH registry.^4^^,^^6^ Median and quartile values from the literature were converted to mean ± SD as previously described.^9^ For estimates, 95% Wald CIs were calculated for means and 95% Wilson-intervals for proportions. Differences between Zurich and registry data were expressed as mean and proportion differences, with 95% CI calculated using the square-and-add method.

Hemodynamic and functional parameters such as mean pulmonary artery pressure (mPAP), PVR, cardiac output (CO), cardiac index, transfer factor of the lung for carbon monoxide, forced expiratory volume in 1 second, and 6-minute walk test (6MWT) from baseline to follow-up were compared using paired *t* tests. Oxygen use was analyzed with the McNemar test, and New York Heart Association (NYHA) functional class changes were analyzed with Kornbrot rank difference test. Analysis of covariance was used to compare values across the 3 time points—baseline, postoperative, and follow-up—using the change from baseline to postoperative as predictor for the follow-up value and adjusting for the baseline value as a covariate. Risk factor analysis was conducted using logistic regression to assess preoperative clinical variables (age, body mass index [BMI], Jamieson), laboratory variables (NT-pro-B-type natriuretic peptide [NT-proBNP], red cell distribution width [RDW], fibrinogen, anti-cardiolipin-immunoglobulin G (IgG), anti-beta2-glycoprotein-1-IgG), and hemodynamic variables (ie, mPAP and PVR) as prognosticators for complications, hemodynamic improvement, and need for multimodal treatment. Univariable regressions were applied. Heavily skewed laboratory values were log-transformed. To control for multiple comparisons, *P* values were adjusted using the Benjamini-Hochberg procedure. All analyses were performed using R (R Foundation for Statistical Computing).

## Results

### Study Population

One hundred forty-one patients with CTEPH underwent PEA at our institution between January 2015 and December 2024. The cohort included 85 (60.3%) male patients, with a median age of 62 years (51-71 years) and a mean BMI of 27.58 ± 5.39. Forty-three (32.1%) patients were NYHA functional class II, and 78 (58.2%) were NYHA functional class III. Preoperatively, 44 (31.2%) patients required oxygen therapy and 64 (46.0%) PH medication. The most common comorbidities were obesity (n = 32 [22.7%]), hyperlipidemia (n = 241 [7.0%]), sleep apnea (n = 22 [15.6%]), chronic renal disease (n = 19 [13.5%]), and chronic obstructive pulmonary disease (n = 18 [12.8%]) (Table E1). The predominant Jamieson classification was type III on both the right (n = 42 [33.9%]) and left side (n = 58 [47.2%]).

### Perioperative Outcomes

**Figure 1:**
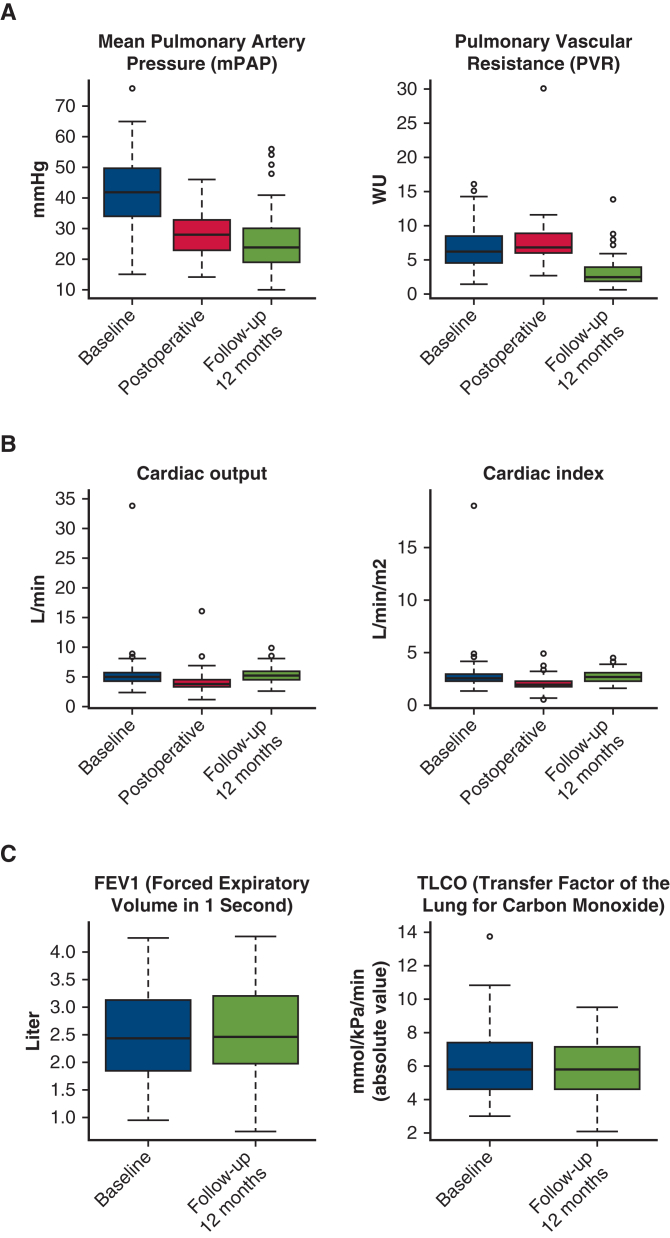

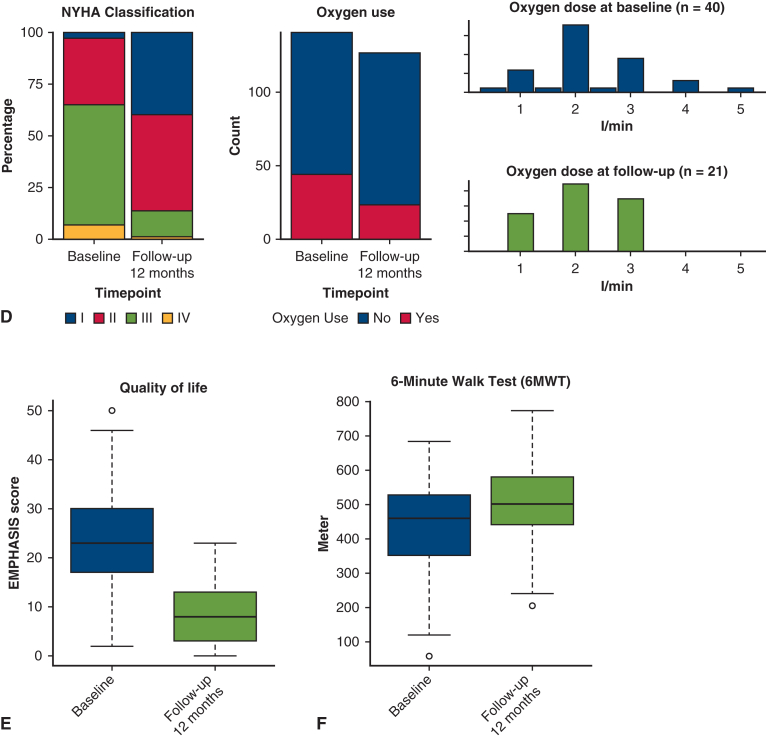
A, mean pulmonary arterial pressure (*mPAP*) and pulmonary vascular resistance (*PVR*). B, Cardiac output (*CO*) and cardiac index (*CI*) at time points: preoperatively (baseline), postoperatively, and follow-up. C, forced expiratory volume in 1 second (*FEV1*) and transfer factor of the lung for carbon monoxide (*TLCO*) preoperatively and at follow-up. D, New York Heart Association functional class (*NYHA*), oxygen use, and dose preoperatively and at follow-up. E, Quality of life (*QoL*) preoperatively and at follow-up. F, Six-minute-walk-test (*6MWT*) preoperatively and at follow-up. Box-and-whisker plots: lower and upper edges mark 25th and 75th percentiles, *middle line* shows median, *whiskers* show minimum and maximum values, and *dots* indicate outliers.

The mean circulatory arrest time was 43.47 ± 12.47 minutes. We observed significant reductions in mPAP (mean difference, 12.9 mm Hg; *P* < .0001), CO (mean difference, 1.1 mL/min; *P* = .032), and cardiac index (mean difference, 0.7 L/min/m^2^; *P* = .011) immediately after PEA (Figure 1). The most common postoperative complications classified as Clavien-Dindo ≥III were atrial fibrillation (n = 14 [9.9%]), hemothorax (n = 12 [8.5%]), and acute renal failure requiring dialysis (n = 10 [7.1%]) (Table 1). The median hospitalization time was 14 days (range, 11-21 days). In-hospital mortality rate was 2.8% (n = 4), and no additional deaths occurred within 90 days postoperatively, resulting in a 90-day mortality rate of 2.8% (Table 2).

**Table 1:** Postoperative complications classified as Clavien-Dindo grade ≥III of patients with chronic thromboembolic pulmonary hypertension (CTEPH) undergoing pulmonary endarterectomy (PEA)

Postoperative complications	Patients		Missing (%)
	n	%	
Right ventricular failure			0.0
Clavien-Dindo IIIa	3	2.1	
Clavien-Dindo V	1	0.7	
Atrial fibrillation			
Clavien-Dindo IIIa	14	9.9	0.0
AVNRT			
Clavien-Dindo IIIa	1	0.7	0.0
Pericardial effusion			
Clavien-Dindo IIIb	7	4.9	0.0
Cardiac tamponade			
Clavien-Dindo IIIa	1	0.7	0.0
Clavien-Dindo IIIb	5	3.5	0.0
Lung reperfusion edema			
Clavien-Dindo IIIa	1	0.7	0.0
Ventilator-associated pneumonia			
Clavien-Dindo IIIa	3	2.1	0.0
Clavien-Dindo IIIb	1	0.7	0.0
Intrapulmonary (airway) bleeding			
Clavien-Dindo IIIa	3	2.1	0.0
ARDS			
Clavien-Dindo IIIb	1	0.7	0.0
Respiratory failure			
Clavien-Dindo IIIa	1	0.7	0.0
Clavien-Dindo IIIb	4	2.8	0.0
Hemothorax			
Clavien-Dindo IIIb	12	8.5	0.0
Pneumothorax			
Clavien-Dindo IIIb	6	4.2	0.0
Pulmonary embolism			
Clavien-Dindo IVb	1	0.7	0.0
Acute renal failure			
Clavien-Dindo IVa	10	7.0	0.0
Ischemic bowel			
Clavien-Dindo IIIb	1	0.7	0.0
Instability of the sternum			
Clavien-Dindo IIIb	2	1.4	0.0
Wound infection			
Clavien-Dindo IIIb	2	1.4	0.0
Patients with complications of Clavien-Dindo III or IV	38	31.9	0.0

*AVNRT*, Atrioventricular nodal reentry tachycardia; *ARDS*, acute respiratory distress syndrome.

**Table 2:** Baseline characteristics, perioperative outcomes, and survival outcomes of patients with chronic thromboembolic pulmonary hypertension (CTEPH) undergoing pulmonary endarterectomy (PEA) (N = 141)

	Patients with CTEPH undergoing PEA	Missing (%)
Baseline		
Age (y)	62 (51-71)	0.0
Male sex	85 (60.3)	0.0
New York Heart Association functional class		5.0
I	4 (3.0)	
II	43 (32.1)	
III	78 (58.2)	
IV	9 (6.7)	
Body mass index	27.58 ± 5.39	0.0
Oxygen usage	44 (31.2)	0.0
6MWT distance (m)	460.00 (351.00-530.00)	2.8
mPAP (mm Hg)	41.96 ± 10.61	5.7
PVR (WU)	6.70 ± 3.16	7.8
FEV1 (%)	81.63 ± 20.09	25.5
Perioperative		
PAH medication	64 (46.0)	1.4
Anticoagulation	138 (97.9)	0.0
Jamieson, left		12.8
I	11 (8.9)	
II	40 (32.5)	
III	58 (47.2)	
IV	14 (11.4)	
Jamieson, right		12.1
I	33 (26.6)	
II	39 (31.5)	
III	42 (33.9)	
IV	10 (8.1)	
Surgery duration (min)	423.43 ± 66.87	0.0
Circulatory arrest time (min)	43.47 ± 12.47	0.0
Aortic crossclamp time (min)	110.65 ± 35.36	0.0
CPB time (min)	299.58 ± 53.66	0.0
Outcome		
Postoperative ECMO	10 (8.3)	0.7
Hospitalization duration (d)	14.00 (11.00-21.00)	0.0
ICU duration (d)	5.00 (3.00-9.00)	0.0
In-hospital mortality	4 (2.8)	0.0
30-d mortality	4 (2.8)	0.0
90-d mortality	4 (2.8)	0.0
Patient status∗		0.0
Alive	128 (90.78)	
Dead	13 (9.22)	
Follow-up after PEA (y)	3.9 (1.4-5.6)	0.0
Multimodal treatment	21 (16.7)	10.6

Values are presented as mean ± standard deviation, median (interquartile range), or n (%). *6MWT*, 6-Minute-walk-test; *mPAP*, mean pulmonary arterial pressure; *PVR*, pulmonary vascular resistance; *FEV1*, forced expiratory volume in 1 second; *PAH*, pulmonary arterial hypertension; *CPB*, cardiopulmonary bypass; *ECMO*, extracorporeal membrane oxygenation; *ICU*, intensive care unit.

∗ March 17, 2025.

### Long-term Outcomes

**Figure 2:**
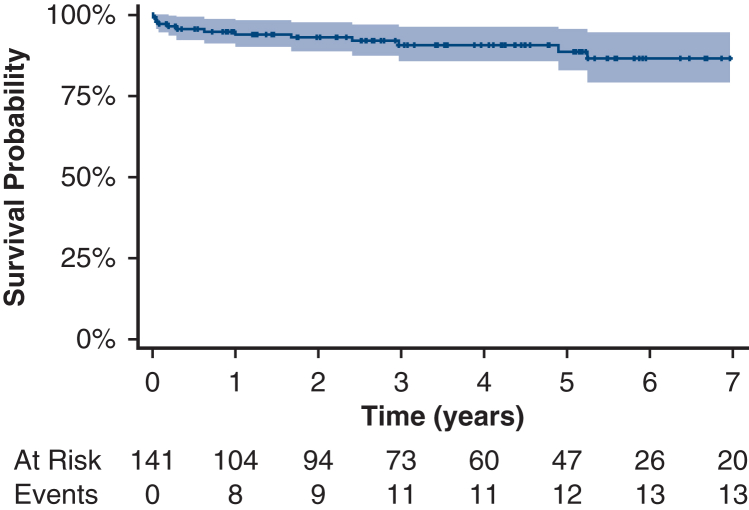
Kaplan-Meier curve displaying postoperative overall survival, including table with numbers at risk for patients with chronic thromboembolic pulmonary hypertension undergoing pulmonary endarterectomy. 95% CI.

At 1-year follow-up, we observed improvements in mPAP (decrease of 16.7 mm Hg; *P* < .0001), PVR (decrease of 3.7 WU; *P* < .0001), 6MWT (increase of 68.8 m; *P* < .0001), decrease in oxygen requirement (χ^2^ = 6.3%; *P* = .018), improvement in NYHA classification (rank difference statistic = −8%; *P* < .0001), and QoL (improvement of 13.7 points; *P* = .004) (Figure 1, Table E2). No significant changes were noted in forced expiratory volume in 1 second, transfer factor of the lung for carbon monoxide, CO, or cardiac index (Table 3). Ninety percent survival was 4.9 years, with a 95% CI from 1.0 to >9.6 years, and median follow-up time 3.9 years (1.4-5.6 years) (Figure 2). The 1-, 2-, and 5-year survival rates were 94%, 93%, and 89%, respectively. At time of analysis, 128 patients (91.0%) were alive.

**Table 3:** Hemodynamic and pulmonary parameters compared at baseline and follow-up using paired *t* tests∗

Variable	n	MeanDiff	95% CI	*P* value	Adjusted *P* value
mPAP	94	16.628	14.196 to 19.059	<.0001	<.0001
PVR	91	3.702	3.008 to 4.395	<.0001	<.0001
CO	91	0.042	−0.659 to 0.743	.91	.91
Cardiac in dex	87	0.063	−0.33 to 0.455	.75	.86
TLCO	32	0.071	−0.417 to 0.56	.77	.86
FEV1	67	0.011	−0.07 to 0.091	.79	.86
6MWT	107	−68.794	−85.449 to −52.14	<.0001	<.0001
QoL	15	13.733	6.087 to 21.379	.002	.004

*MeanDiff*, Mean difference; *mPAP*, mean pulmonary arterial pressure; *PVR*, pulmonary vascular resistance; *CO*, cardiac output; *TLCO*, transfer factor of the lung for carbon monoxide; *FEV1*, forced expiratory volume in 1 second; *6MWT*, 6-minute-walk-test; *QoL*, quality of life.

∗ Difference is calculated as baseline minus follow-up. *P* values and adjusted *P* values for multiple comparisons are reported using Benjamini-Hochberg procedure.

### Prognosticators

Univariable logistic regression results evaluating age, BMI, mPAP, PVR, and preoperative laboratory markers as factors potentially associated with developing postoperative complications (Clavien-Dindo ≥III) are shown in Table 4. Jamieson IV (odds ratio, 4.2; *P* = .039) and NT-proBNP levels (odds ratio, 1.5; *P* = .039) were associated with postoperative complications, with higher NT-proBNP values indicating increased risk. No associations were found for age, BMI, mPAP, PVR, RDW, fibrinogen, anti-cardiolipin-IgG, or anti-beta2-glycoprotein-1-IgG.

**Table 4:** Univariable logistic regression for postoperative complications (Clavien-Dindo ≥III) and multimodal treatment (pulmonary hypertension medication and/or balloon pulmonary angioplasty after pulmonary endarterectomy)∗

Explanatory variable of interest	Odds ratio	95% CI	*P* value	Adjusted *P* value
Postoperative complication				
Age	1.03	1-1.06	.057	.23
BMI	0.98	0.91-1.05	.49	.54
mPAP	1.01	0.97-1.05	.54	.54
PVR	1.05	0.93-1.19	.41	.54
Jamieson IV	4.22	1.38-13.26	.011	.039
NT-proBNP (log)	1.47	1.1-2.01	.013	.039
RDW (log)	3.95	0.24-59.18	.32	.35
Anti-cardiolipin-IgG (log)	0.78	0.43-1.28	.35	.35
Anti-beta2-GP1-IgG (log)	0.66	0.32-1.12	.19	.28
Fibrinogen	1.56	0.83-3.01	.17	.28
Multimodal treatment				
Age	0.98	0.95-1.01	.22	.59
BMI	1.03	0.95-1.12	.40	.59
mPAP	1.02	0.98-1.06	.44	.59
PVR	1.00	0.87-1.15	.97	.97
Jamieson IV	3.88	1.05-13.46	.034	.14
NT-proBNP (log)	1.06	0.77-1.49	.71	.71
RDW (log)	15.23	0.73-313.4	.071	.14
Anti-cardiolipin-IgG (log)	0.78	0.44-1.28	.35	.42
Anti-beta2-GP1-IgG (log)	0.51	0.21-0.94	.068	.14
Fibrinogen	0.55	0.22-1.31	.19	.28

*BMI*, Body mass index; *mPAP*, mean pulmonary arterial pressure; *PVR*, pulmonary vascular resistance; *NT-proBNP*, NT-pro-B-type natriuretic peptide; *RDW*, red cell distribution width; *IgG*, immunoglobulin G; *GP1*, glycoprotein 1.

∗ We report *P* values and adjusted *P* values for multiple comparisons using Benjamini-Hochberg procedure.

A separate logistic regression assessed preoperative laboratory values as prognosticators for the need for multimodal treatment. No evidence was found for an association between age, BMI, mPAP, PVR, Jamieson IV, NT-proBNP, RDW, fibrinogen, anti-cardiolipin-IgG, or anti-beta2-glycoprotein-1-IgG and the need for postoperative PH medication or BPA.

Another model analyzed the effect of changes in hemodynamic parameters from baseline to immediate postoperative values on long-term hemodynamic outcomes. Reductions in mPAP (Lin coefficient = 0.7 mm Hg; *P* < .0001) and PVR (Lin coefficient = 0.4 WU; *P* < .0001) were significantly associated with their respective values at 1-year-follow-up. Smaller reductions were linked to higher mPAP and PVR. No such association was observed for CO or cardiac index (Table E3).

### Benchmark Analysis

**Figure 3:**
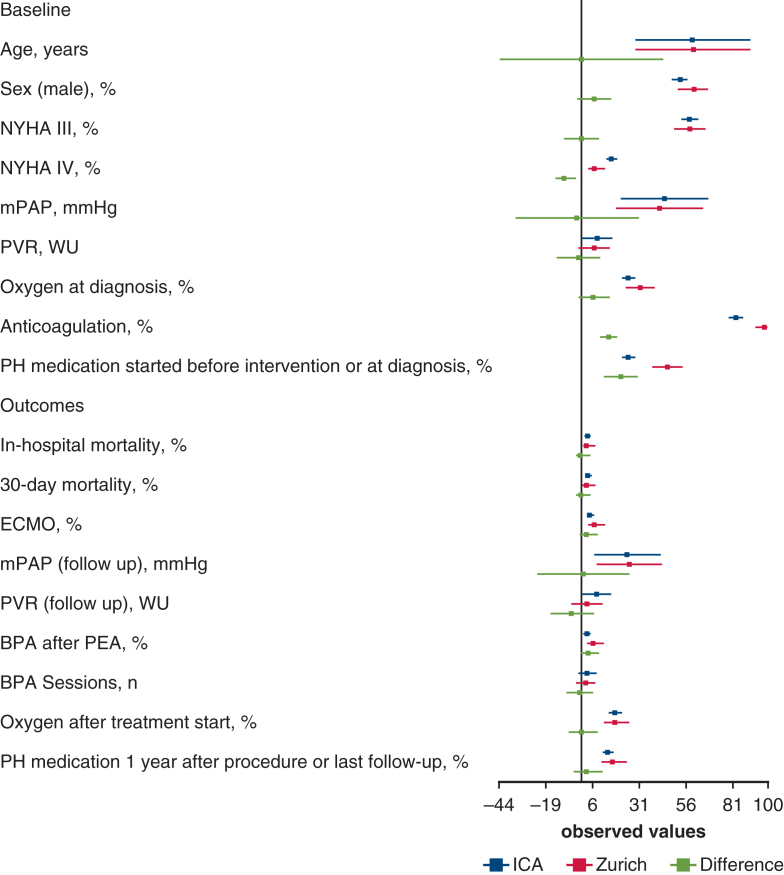
Key parameters of the international chronic thromboembolic pulmonary hypertension association registry^4^ were used in a benchmark analysis with data from the Zurich cohort (Table E4). For continuous variables, means with 95% Wald CI are displayed; for categorical variables, percentages with 95% Wilson CI are shown. Differences in means and proportions are presented with corresponding CI. *NYHA*, New York Heart Association; *mPAP*, mean pulmonary arterial pressure; *PVR*, pulmonary vascular resistance; *WU*, Wood units; *PH*, pulmonary hypertension; *ECMO*, extracorporeal membrane oxygenation; *BPA*, balloon pulmonary angioplasty; *PEA*, pulmonary endarterectomy.

A comparison of our data with the International CTEPH Registry revealed overall comparable baseline and outcome parameters, including age, sex, NYHA, pre- and postoperative hemodynamics, and mortality (Figure 3).^4^^,^^6^ Notable preoperative differences included a lower proportion of patients with NYHA functional class IV in the Zurich cohort (6.7% vs 16.0%) and a higher use of preoperative PH medication (46% vs 25%). Additionally, 97.9% of our patients received preoperative anticoagulation, compared with 83% in the registry. Postoperative PH medication use was similar between groups. However, the rate of BPA following PEA was slightly higher in our cohort (6.4%) than in the registry (2.6%).^6^

### Learning Curve

**Figure E1:**
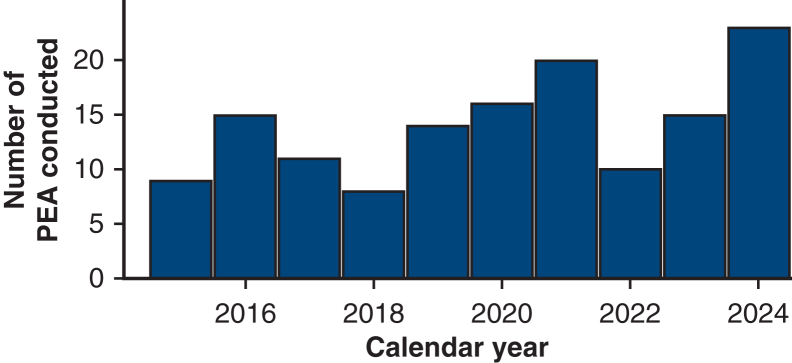
Number of pulmonary endarterectomies (*PEAs*) performed over time during the 10-year experience of the PEA program in Zurich.

**Figure E2:**
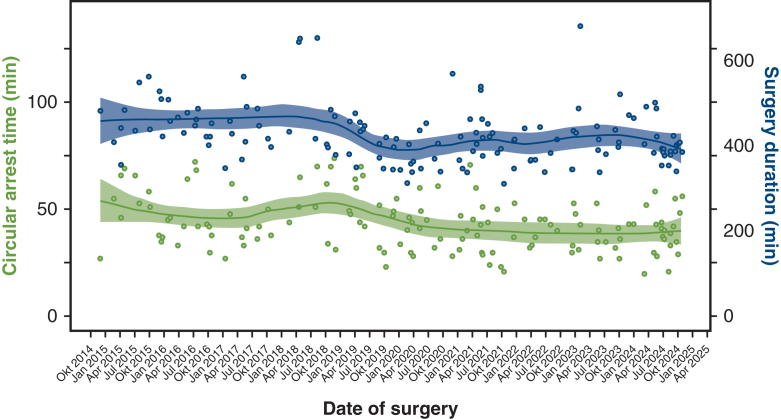
Circulatory arrest time (*left axis*) and surgery duration (*right axis*) for all surgeries done during the 10-year period, measurements and running mean determined with locally estimated scatterplot smoothing smoother with span 0.5, including 95% confidence band.

We report fluctuations in numbers of PEAs over time, with a trend toward an increasing number of surgeries from n = 9 PEAs in 2015 to n = 23 PEAs in 2024 (Figure E1). A decrease in surgery duration and circulatory arrest time is shown in Figure E2. For disease severity and symptoms, no clear trend over the 10 years was observed.

## Discussion

We summarize key outcomes from our 10-year PEA program, established in 2015. Challenges related to Switzerland's small population and limited CTEPH awareness have been addressed through the creation of a national, multidisciplinary CTEPH Board.^7^ Before 2015, the resection rate was just 14%; since the launch of the PEA program and national board, this has steadily increased to 41%.^5^^,^^7^

We analyzed demographic and clinical characteristics of CTEPH patients undergoing PEA at our institution. We observed a significant immediate improvement in mPAP, CO, and cardiac index following PEA. At 12-month follow-up, we recorded further improvements in mPAP, PVR, 6MWT, oxygen requirement, NYHA functional class, and QoL. These findings are consistent with published literature demonstrating hemodynamic and functional improvements after PEA in patients with CTEPH.^10^, ^11^, ^12^ With regard to outcome parameters such as mortality, our results align with those of the International CTEPH Registry. We report in-hospital and 90-day mortality rates of 2.8%, comparable to those seen in high-volume centers, reporting in-hospital mortality rates below 5%,^10^^,^^11^^,^^13^^,^^14^ and 90-day mortality of 3.9%.^10^ Our median hospitalization duration was 14 days (range, 11-21 days), also consistent with durations reported by high-volume centers.^3^

Our baseline characteristics are largely consistent with those reported in the international registry.^4^ We observed no significant differences in baseline demographics or preoperative hemodynamic parameters. However, we noted higher use of preoperative PH medication and anticoagulation therapy in our cohort than in the registry. This may reflect our aggressive multimodal approach, in which we initiate PH-targeted therapy for symptomatic patients (NYHA functional class >I) with PH confirmed by TTE or RHC, and for all patients with PVR >8 WU.^7^ Preoperative anticoagulation is administered strictly in accordance with European Society of Cardiology/European Respiratory Society guidelines.^1^ During postoperative follow-up, PH medication is also prescribed if persistent PH is detected by TTE or RHC and the patient remains symptomatic (NYHA functional class >I). Despite our proactive approach, postoperative PH medication use was comparable between our cohort and the registry. However, we report more patients undergoing BPA after PEA (6.4% vs 2.6%). Previous studies have also documented overlap in the use of PH-targeted therapies among PEA and BPA candidates, with up to one-third of patients receiving PH-targeted medication alongside PEA.^6^^,^^11^

Over the course of 10 years, we observe a decrease in surgery and circulatory arrest duration and a trend toward an increasing number of surgeries per year, which can be attributed to a learning curve during the establishment of the program. Additionally, we adapted our intraoperative management by introducing 5% human albumin into the cardiopulmonary bypass prime and hemodilution solution, as previously described.^15^

Among the analyzed potential prognosticators for postoperative complications, only Jamieson IV and NT-proBNP showed evidence of an association with postoperative complications. This suggests that higher NT-proBNP levels are linked to an increased risk of complications. NT-proBNP has previously been identified as a significant preoperative biomarker for predicting postoperative complications in non-cardiac surgical procedures, with elevated levels consistently associated with increased postoperative mortality and adverse cardiovascular events.^16^^,^^17^ In patients with CTEPH, NT-proBNP has been shown to predict postoperative morbidity and mortality.^18^ NT-proBNP may not only reflect the hemodynamic severity of CTEPH but also assist in evaluating perioperative risk and therapeutic effect of PEA.^19^ We assessed various clinical and laboratory factors for prognostic value for postoperative multimodal treatment but found no associations.

We found strong evidence that the initial change in mPAP and PVR (baseline to postoperative) influences 1-year hemodynamic outcomes. A greater immediate decrease postoperatively was associated with better long-term hemodynamic results. This finding raises the question of whether patients with smaller reductions should be more closely monitored postoperatively and considered for multimodal therapy earlier. A limited immediate postoperative decrease in mPAP and PVR may indicate a higher likelihood of residual PH and need for additional treatment.

### Limitations

This study has several limitations. Although data collection was prospective, the analysis is retrospective in nature. Being a single-center study, the generalizability of the findings is limited. Because cohort data were used, the timing of measurements was not fixed but occurred within defined ranges. Narrowing these ranges could help reduce bias, but may also increase the number of missing values, introducing another potential source of bias. Adequate risk adjustment could not be performed due to the small patient cohort and number of events. Further efforts are needed to better understand preoperative prognosticators for postoperative complications and multimodal therapy.

## Conclusions

The establishment of a PEA program in Switzerland has ensured timely, gold standard treatment for patients with CTEPH. Despite being a small-volume program, outcomes in terms of short- and long-term hemodynamic and functional improvements, as well as survival, are comparable to those reported by high-volume centers and the International CTEPH Registry. Preoperative NT-proBNP levels and Jamieson IV appear to be associated with postoperative complications. Additionally, a greater immediate postoperative decrease in mPAP and PVR is associated with better long-term hemodynamic outcomes. Patients with smaller initial reductions should be closely monitored and may benefit from early consideration for multimodal therapy.

### Webcast

You can watch a Webcast of this AATS meeting presentation by going to: https://www.aats.org/resources/10-years-pulmonary-endarterect-9445.

**Figure:**
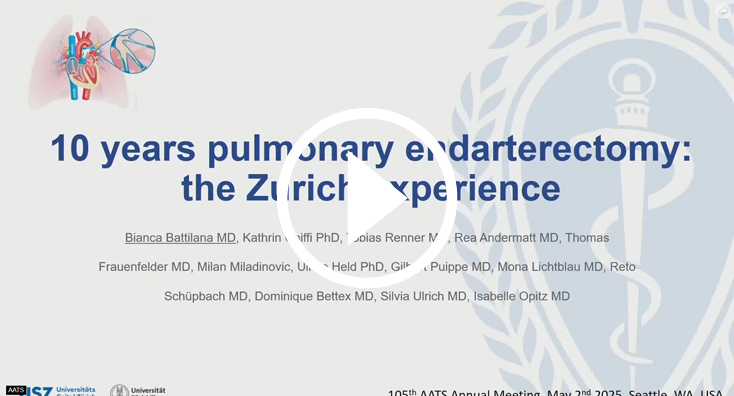

### Audio

You can listen to the discussion audio of this article by going to the supplementary material section below.

## Conflict of Interest Statement

Dr Opitz reports relationships with Roche (institutional grant), Roche Genentech (steering committee), AstraZeneca (advisory board), MSD (advisory board), BMS (advisory board), Medtronic (institutional grant and advisory board), Intuitive (proctorship and speaker's fee), Sanofi (speaker's fee), Regeneron (advisory board), XVIVO (institutional grant), and Siemens (speaker's fee). Dr Ulrich reports research grants (Swiss National Science Foundation, Zurich and Swiss Lung League, EMDO foundation), grants, travel support, and consultancy fees (Orpha Swiss, Janssen SA, MSD SA, Gebro SA, Ideogen), all unrelated to the present work. Dr Lictblau reports travel support and consultancy fees (Orpha Swiss, Johnson & Johson, MSD, and Gebro), unrelated to the present work. All other authors reported no conflicts of interest.

The *Journal* policy requires editors and reviewers to disclose conflicts of interest and to decline handling or reviewing manuscripts for which they may have a conflict of interest. The editors and reviewers of this article have no conflicts of interest.

## Figures and Tables

**Table E1:** Comorbidities of patients with chronic thromboembolic pulmonary hypertension undergoing pulmonary endarterectomy

Type of comorbidity	Patients		Missing (%)
	n	%	
Coagulation/thrombophilic disorder	21	14.8	0
Chronic venous insufficiency	6	4.3	0
Coronary artery disease	11	7.8	0
Congestive heart failure	9	6.4	0
Coronary heart disease	8	5.7	0
Left ventricular diastolic dysfunction	1	0.7	0
Patent foramen ovale	13	9.2	0
Rhythmical heart disease	12	8.5	0
Valvular heart disease	2	1.4	0
Chronic obstructive pulmonary disease	18	12.8	0
Interstitial lung disease	3	2.1	0
Sleep apnea	22	15.6	0
Diabetes mellitus	15	10.6	0
Hyperlipidemia	24	17.0	0
Obesity	32	22.7	0
Hyperthyroidism	2	1.4	0
Hypothyroidism	15	10.6	0
Chronic renal disease	19	13.5	0
Inflammatory bowel disease	1	0.7	0
Splenectomy	1	0.7	0
History of cancer	13	9.2	0
Other	116	82.3	0

**Table E2:** McNemar test for oxygen therapy (yes or no) at baseline and follow-up and rank difference test for New York Heart Association (NYHA) classification at baseline and follow-up

Variable	n	Statistic	*P* value	Adjusted *P* value
Oxygen therapy	127	6.323	.012	.018
NYHA	114	−7.995	<.0001	<.0001

**Table E3:** Logistic regression showing the influence of the change from baseline to postoperative values on follow-up outcomes, including estimates with 95% CI, *P* values, and Benjamini-Hochberg-adjusted *P* values

Variable	n	Estimate	95% CI	*P* value	Adjusted *P* value
mPAP	71	0.677	0.423 to 0.931	<.0001	<.0001
PVR	51	0.401	0.208 to 0.595	<.0001	<.0001
CO	51	0.065	−0.082 to 0.212	.39	.52
Cardiac index	50	−0.042	−0.283 to 0.198	.73	.73

*mPAP*, Mean pulmonary arterial pressure; *PVR*, pulmonary vascular resistance; *CO*, cardiac output.

**Table E4:** Key parameters from data from the International Chronic Thromboembolic Pulmonary Hypertension Registry^4^ that were used for the benchmark analysis

Variable	Mean/Prop	95% CI
Baseline parameter		
Age (y)	59.7	29.1 to 90.3
Male sex (%)	53.1	49.1 to 57
NYHA class III (%)	58.0	54 to 61.9
NYHA class IV (%)	16.0	13.3 to 19.2
mPAP (mm Hg)	44.3	21 to 67.6
PVR (WU)	8.4	0.3 to 16.5
Oxygen at diagnosis (%)	25.0	21.7 to 28.6
Anticoagulation (%)	83.0	79.8 to 85.8
PH medication started before intervention or at diagnosis (%)	25.0	21.7 to 28.6
Outcome parameters		
In-hospital mortality (%)	3.1	2 to 4.9
30-d mortality (%)	3.1	2 to 4.9
ECMO (%)	4.5	3.1 to 6.4
mPAP at follow-up (mm Hg)	24.7	7.2 to 42.1
PVR (follow up) (WU)	8.1	0 to 16.2
BPA after PEA (%)	2.6	1.6 to 4.3
BPA sessions (n)	3.2	−1.6 to 7.9
Oxygen after treatment start (%)	18.0	15.2 to 21.3
PH medication 1 y after procedure or last follow-up (%)	14.0	11.5 to 1

Values are presented as mean or proportions (Mean/Prop) and 95% Wald or Wilson 95% CI. *NYHA*, New York Heart Association; *mPAP*, mean pulmonary arterial pressure; *PVR*, pulmonary vascular resistance; *PH*, pulmonary hypertension; *ECMO*, extracorporeal membrane oxygenation; *BPA*, balloon pulmonary angioplasty; *PEA*, pulmonary endarterectomy.

## Supplementary Data

### Audio

Audio 1 This is an audio recording of the presentation of the associated abstract and discussion. The abstract presentation ends at 7:22 and the discussion begins at 7:25.

Video 1 Video available at: https://www.jtcvs.org/article/S2666-2736(25)00434-6/fulltext.
